# Retraction: Infectious clone construction and pathogenicity confirmation of Cotton leaf curl Multan virus (CLCuMuV), Ramie mosaic virus (RamV) and Corchorus yellow vein Vietnam virus (CoYVV) by southern blot analysis

**DOI:** 10.1371/journal.pone.0286780

**Published:** 2023-06-14

**Authors:** The PLOS ONE Editors

The *PLOS ONE* Editors retract this article [1] because it was identified as one of a series of submissions for which we have concerns about authorship, competing interests, and peer review. We regret that the issues were not addressed prior to the article’s publication.

Additionally, after this article was published, it was noted that the fourth author’s name was spelled incorrectly. At the time of retraction, this article was republished to provide the correct name.

AMAS agreed with the retraction. AMES and SSA did not agree with the retraction. MA, SSA and MAAA either did not respond directly or could not be reached.
